# Heterophyllin: A New Adenia Toxic Lectin with Peculiar Biological Properties

**DOI:** 10.3390/toxins16010001

**Published:** 2023-12-19

**Authors:** Massimo Bortolotti, Francesco Biscotti, Andrea Zanello, Letizia Polito, Andrea Bolognesi

**Affiliations:** Department of Medical and Surgical Sciences-DIMEC, General Pathology Section, Alma Mater Studiorum, University of Bologna, Via San Giacomo 14, 40126 Bologna, Italy; massimo.bortolotti2@unibo.it (M.B.); francesco.biscotti2@unibo.it (F.B.); andreazanello86@gmail.com (A.Z.); andrea.bolognesi@unibo.it (A.B.)

**Keywords:** Adenia, cancer therapy, lectins, plant toxins, ribosome-inactivating proteins, rRNA N-glycosylase, ricin

## Abstract

Ribosome-inactivating proteins (RIPs) are plant toxins that were identified for their ability to irreversibly damage ribosomes, thereby causing arrest of protein synthesis and induction of cell death. The RIPs purified from Adenia plants are the most potent ones. Here, we describe a novel toxic lectin from *Adenia heterophylla* caudex, which has been named heterophyllin. Heterophyllin shows the enzymatic and lectin properties of type 2 RIPs. Interestingly, in immunoreactivity experiments, heterophyllin poorly cross-reacts with sera against all other tested RIPs. The cytotoxic effects and death pathways triggered by heterophyllin were investigated in three human-derived cell lines: NB100, T24, and MCF7, and compared to ricin, the most known and studied type 2 RIP. Heterophyllin was able to completely abolish cell viability at nM concentration. A strong induction of apoptosis, but not necrosis, and the involvement of oxidative stress and necroptosis were observed in all the tested cell lines. Therefore, the enzymatic, immunological, and biological activities of heterophyllin make it an interesting molecule, worthy of further in-depth analysis to verify its possible pharmacological application.

## 1. Introduction

The Adenia genus (Passifloraceae) consists of about 100 plant species restricted to the Old-World tropical and subtropical areas. Some Adenia species are utilized in traditional African medicine [1] and are known to contain lethal toxins [2], identified as ribosome-inactivating proteins (RIPs), toxic enzymes broadly distributed in different plant genera [3,4].

Regarding their structure, RIPs are mainly divided into type 1, 30 kDa-single chain proteins with enzymatic activity, and type 2, 60 kDa-double chain proteins consisting of an enzymatically active A-chain linked to a lectin B-chain that binds to sugar moieties on the cell surface, facilitating the endocytosis of the toxin. Many type 2 RIPs are potent toxins [5,6,7] and among them, ricin, from the seeds of *Ricinus communis* L., is the most studied RIP and it is known both for its biomedical applications and for its use as a chemical weapon for bio-crimes [5,8,9].

RIPs are classified as rRNA N-glycosylases (EC 3.2.2.22); in fact, they can remove a specific adenine in a GAGA sequence on 28S rRNA, thus blocking the binding of elongation factors to the 60S subunit of the ribosome and causing the consequent arrest of translation [10,11]. Besides rRNA, RIPs possess N-glycosylase activity on other substrates, such as genomic DNA, mRNA, tRNA, viral nucleic acids, polyA, and poly (ADP-ribose) sequences. Therefore, RIP activity was also termed polynucleotide:adenosine glycosylase (PNAG) [12,13]. Being able to act on several independent substrates, RIPs are capable of inducing different death pathways in intoxicated cells. RIPs are hence able to kill cells even if they have alterations in proliferative or apoptotic mechanisms, thus making it impossible to select RIP-resistant mutants [14,15,16,17].

The type 2 RIPs so far known belong to a few plant genera [18,19]; among them, the most known cytotoxic RIPs are abrin, ricin, and several RIPs purified from Adenia plants. Modeccin and volkensin, from *Adenia* (Modecca) *digitata* (Harv.) Engl and *Adenia volkensii* Harms, respectively, were the first type 2 RIPs purified and characterized from Adenia plants [20,21,22,23,24,25]. Moreover, three highly cytotoxic type 2 RIPs from *Adenia lanceolata* Engl., *Adenia stenodactyla* Harms, and *Adenia kirkii* (Mast) Engl. were purified and named lanceolin, stenodactylin, and kirkiin, respectively [26,27].

The Adenia RIP potency can depend on several characteristics, such as high affinity in binding cells, proper endocytosis and intracellular trafficking, low proteolytic susceptibility, and high stability [28,29]. Moreover, Adenia toxins can have important potential medical and biotechnological applications in neurophysiology and pain treatment, because of their ability to be retrogradely transported along peripheral nerves and in the central nervous system [30,31]. Recently, stenodactylin has been evaluated for its use in the local-regional treatment of strabismus and muscular dystonia [32]. Besides their use as native proteins, the A-chain of type 2 RIPs can be used as a toxic payload of immunoconjugates for several clinical applications, mainly for cancer therapy [33,34,35].

The peculiar features of Adenia toxins prompted us to investigate whether other plants belonging to the Adenia genus produce type 2 RIPs. In this work, a new toxic type 2 RIP, hereinafter named heterophyllin, was isolated from *Adenia heterophylla* (Blume) Koord. We describe the purification protocol and a preliminary analysis of heterophyllin biochemical, enzymatic, immunological, and cytotoxic properties.

## 2. Results

### 2.1. Purification and Characterization of Adenia heterophylla Lectin

**Figure 1:**
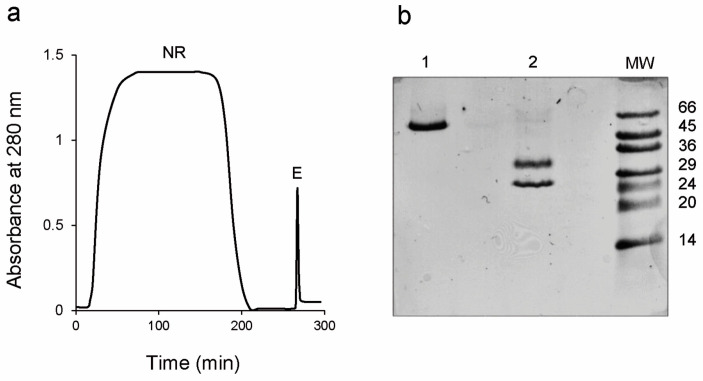
Purification of *Adenia heterophylla* lectin. (**a**) Chromatography on acid treated-Sepharose CL-6B column of *A. heterophylla* extracts. The first peak (NR) corresponds to not retained proteins. The second peak (E) corresponds to proteins eluted with 0.5 M galactose in saline phosphate buffer. (**b**) SDS-PAGE analysis of the eluted lectins under non-reducing (lane 1) and reducing (lane 2) conditions. The electrophoresis was carried out on a 4–15% gradient gel SDS-PAGE. Standard molecular weights (MW) are expressed in kDa.

The crude extract from *A. heterophylla* caudex was purified by chromatography on an acid-treated Sepharose CL-6B column. As described in the materials and methods section, this step allows the purification of the lectins in the crude extract. As can be seen in the chromatogram reported in Figure 1a, the first peak corresponds to the proteins that were not retained (NR) by the resin. After elution with 0.5 M galactose, a sharp peak was eluted (E). The eluted peak was analyzed on a 4–15% gradient gel SDS-PAGE (Figure 1b). The analysis showed a single band of about 60 kDa under non-reducing conditions (lane 1) and two bands of about 25 and 33 kDa under reducing conditions (lane 2). The yield was 5.6 mg of purified lectin (Table 1), from 460 g of fresh tissue.

### 2.2. Enzymatic Activities of the Lectin from A. heterophylla Caudex

**Figure 2:**
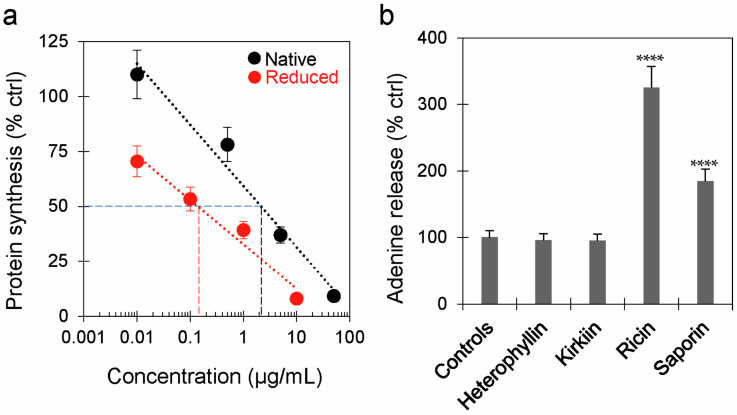
Enzymatic activities of heterophyllin. (**a**) rRNA N-glycosylase activity of heterophyllin was indirectly evaluated using a cell-free rabbit reticulocyte lysate system. Protein synthesis was evaluated under reducing (Reduced) and non-reducing conditions (Native). The concentration causing 50% inhibition of protein synthesis (IC_50_) was calculated by linear regression analysis. (**b**) Polynucleotide:adenosine glycosylase activity of heterophyllin, kirkiin, ricin, and the type 1 RIP saporin on herring sperm DNA. The amount of released adenines was determined by measuring spectrophotometrically the absorbance at 260 nm. The results are expressed as means ± SD of three independent experiments, each conducted in duplicate. Data were analyzed by the Mann–Whitney U test (confidence range 95%; **** *p* < 0.0001 RIPs versus controls).

The ability of the purified lectin to inhibit protein synthesis was assayed in a cell-free rabbit reticulocyte lysate system (Table 1 and Figure 2a). The protein showed high enzymatic activity, with concentrations inhibiting 50% of protein synthesis (IC_50_) of 2.11 µg/mL and 0.142 µg/mL for the non-reduced and reduced protein, respectively. The purification yield, calculated based on the total activity of the crude extract, was 55.7%. Moreover, *A. heterophylla* lectin showed agglutinating activity on human erythrocytes, with a minimum agglutinating concentration of 43.25 µg/mL (Table 1).

This lectin, with a molecular weight of approximately 60 kDa, able to inhibit protein synthesis in a cell-free system and to agglutinate red blood cells, shows all the characteristics of a type 2 RIP, and will henceforth be named heterophyllin.

To evaluate the enzymatic properties of heterophyllin on DNA, the release of adenines from lectin-treated herring sperm DNA was spectrophotometrically determined. As shown in Figure 2b, heterophyllin had PNAG activity not significantly different from untreated controls, similarly, as already reported for kirkiin [27]. In the same experiments, saporin and ricin, used as positive controls, showed significantly higher PNAG activity. Our experiments indicated that heterophyllin had no significant activity on herring sperm DNA.

### 2.3. Immunological Properties of Heterophyllin

**Figure 3:**
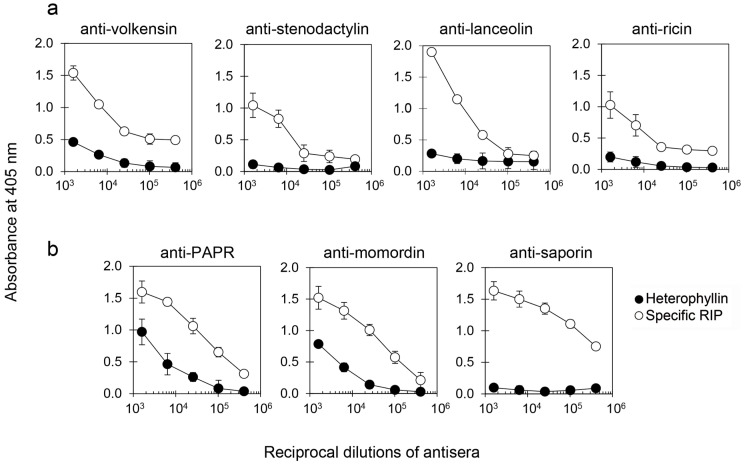
Enzyme-linked immunosorbent assay (ELISA) with (**a**) anti-type 2 and (**b**) anti-type 1 RIP sera. The reactivity curves of heterophyllin (black symbols) and specific RIP (white symbols) towards antisera are expressed as absorbance at 405 nm in function of the reciprocal dilutions of antiserum. The results are expressed as means ± SD of three independent experiments, each conducted in duplicate.

Heterophyllin was tested with sera against other type 2 RIPs, i.e., volkensin, stenodactylin, lanceolin (all from Adenia plants), and ricin to evaluate their cross-reactivity. Heterophyllin poorly reacted with sera against type 2 RIPs (Figure 3a). Regarding sera against type 1 RIPs, i.e., PAP-R, momordin, and saporin, heterophyllin showed a low cross-reactivity with anti-PAP-R and anti-momordin sera, while it did not react at all with anti-saporin serum (Figure 3b).

### 2.4. Biological Effects of Heterophyllin on Three Human-Derived Cell Lines

#### 2.4.1. Heterophyllin Was Able to Completely Abolish Cell Viability

The cytotoxic effects of heterophyllin were evaluated, in comparison to ricin, on three human cell lines: the neuroblastoma-derived NB100, the bladder carcinoma-derived T24, and the breast carcinoma-derived MCF7.

**Figure 4:**
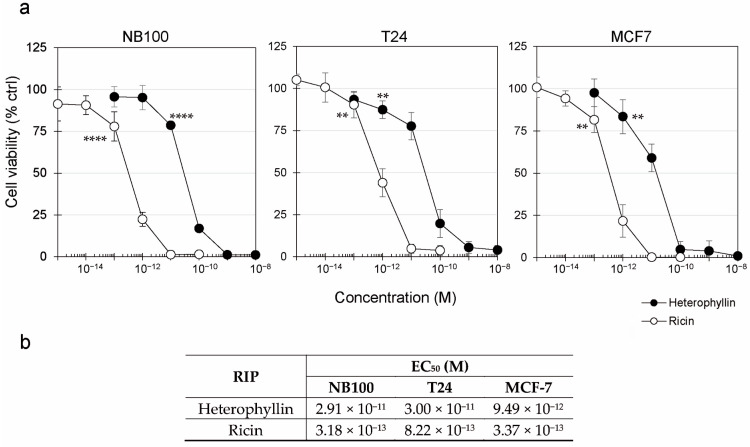
Concentration–response experiments. (**a**) Cells (3 × 10^4^/well) were treated with heterophyllin (●) or ricin (○) for 72 h. Viability was evaluated using a colorimetric assay based on MTS reduction. (**b**) The table reports concentration values that reduce cell viability by 50% (EC_50_), calculated by linear regression analysis. The results are expressed as means ± SD of three independent experiments, in triplicate. The results are reported as a percentage of untreated controls. **** *p* ≤ 0.0001, ** *p* < 0.01, ANOVA/Bonferroni, with Dunnett’s test.

The cell viability was evaluated after 72 h incubation through concentration-response experiments, using scalar concentrations of RIPs. In all the tested cell lines, ricin started to significantly kill cells at 10^−13^ M concentration, whereas heterophyllin started to significantly kill cells at 10^−11^ M (NB100) and 10^−12^ M (T24 and MCF7) (Figure 4a). Heterophyllin showed a good ability to reduce cell viability, with an effective concentration of RIP reducing 50% of viability (EC_50_) of about 10^−11^ M for all the tested cell lines (Figure 4b). Complete cell killing was obtained at 10^−9^ M concentration in NB100 and T24 cells, and 10^−10^ M in MCF7 cells. Ricin was more toxic than heterophyllin, showing EC_50_ values of about 10^−13^ M; the complete cell killing was observed at 10^−11^ M concentration in all the tested cell lines.

Overall, the obtained data showed that heterophyllin is about 2-log less toxic than ricin. As heterophyllin and ricin completely killed the cells at 10^−9^ M and 10^−11^ M after 72 h incubation, respectively, these concentrations were chosen for further cytotoxicity experiments carried out at 24 h.

#### 2.4.2. Heterophyllin Induced Apoptosis but Not Necrosis

**Figure 5:**
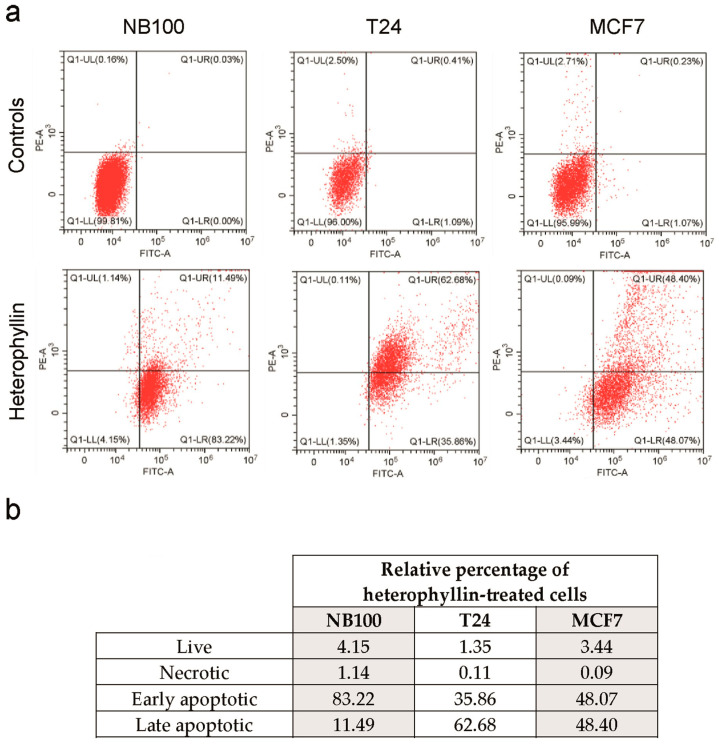
Apoptosis/necrosis evaluation after Annexin V-EGFP/PI staining and flow cytometry analysis. (**a**) NB100, T24, and MCF7 cell lines were cultured in the absence or presence of heterophyllin at the concentration of 10^−9^ M for 24 h. Apoptosis and necrosis were evaluated through flow cytometry analysis. Necrotic cells (PI-positive) are in the upper left quadrant, while apoptotic cells are in the upper (late apoptosis; PI-positive and EGFP-positive) and lower (early apoptosis; PI-negative and EGFP-positive) right quadrants. Representative plots of Annexin V-EGFP (FITC channel)/PI (PE channel) staining of NB100, T24, and MCF7 cells are shown. The plots are representative of two independent experiments, each conducted in triplicate. The table (**b**) reports the percentages of live, necrotic, and early/late apoptotic cells after heterophyllin treatment.

The involvement of apoptosis and necrosis in heterophyllin-treated cells was evaluated through cytofluorimetric analysis, double staining the cells with Annexin V-EGFP/Propidium iodide (PI). After 24 h of intoxication with 10^−9^ M heterophyllin, more than 95% of treated cells were in early/late-stage apoptosis. Heterophyllin did not significantly induce necrosis in treated cells (Figure 5).

#### 2.4.3. Heterophyllin Cytotoxicity Was Strongly Reduced by ROS Scavengers and Cell Death Inhibitors

To determine the role of oxidative stress, apoptosis, and necroptosis in the pathogenesis of the intoxication induced by heterophyllin and ricin, experiments were conducted pre-treating cells for 3 h with the reactive oxygen species (ROS) scavengers, catalase (CAT) and sodium pyruvate (NaPyr), with the pan-caspase inhibitor Z-VAD and with the necroptosis inhibitor necrostatin-1 (NEC). The cells were treated for 2 h with heterophyllin or ricin at the lowest concentration abolishing cell viability in previous carried out experiments at 72 h, i.e., 10^−9^ and 10^−11^ M, respectively, and the cytotoxicity was evaluated after 24 h.

**Figure 6:**
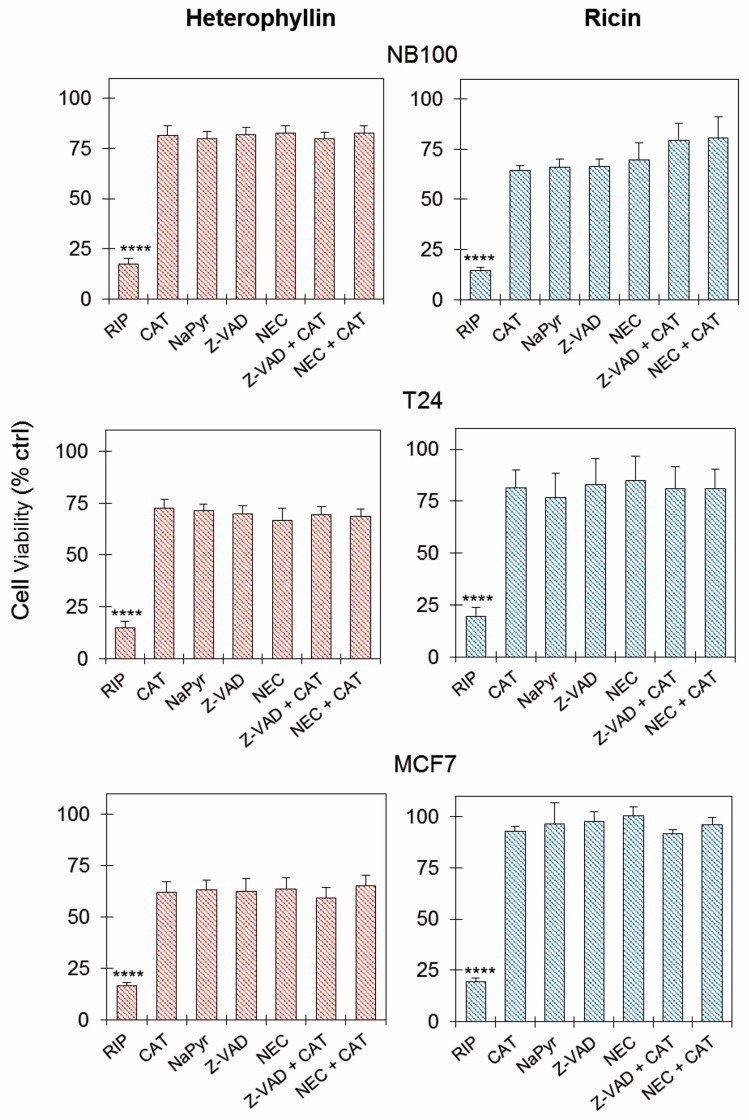
Effect of ROS scavengers and cell death inhibitors on heterophyllin cytotoxicity. Viability of NB100, T24, and MCF7 cells (5 × 10^3^/well), treated for 2 h with 10^−9^ M heterophyllin or 10^−11^ M ricin, without or in presence of 10 U/mL catalase (CAT) or 1 mM sodium pyruvate (NaPyr); 100 μM Z-VAD or 100 μM necrostatin-1 (NEC); combination of CAT plus cell death inhibitors. Scavengers and inhibitors were added 3 h before the RIP treatment. After this, cells were treated for 2 h with RIPs and further incubated for 24 h in a complete medium. The results are expressed as means ± S.D. of three independent experiments, each conducted in triplicate. Data were analyzed by the Mann–Whitney U test. Asterisks indicate the significant difference in each experimental condition between RIPs alone and RIPs plus inhibitors/scavengers. **** *p* < 0.0001.

As shown in Figure 6, both the scavengers and the cell death inhibitors were able to significantly (*p* < 0.0001) increase the survival of heterophyllin- or ricin-treated cells in all the tested cell lines. In NB100 cells, the pre-treatment with scavengers/inhibitors conferred higher protection in heterophyllin-treated cells than in ricin-treated cells. On the contrary, in T24 and MCF7 cells, the pre-treatment with scavengers/inhibitors caused a higher protective effect in ricin-intoxicated cells compared to heterophyllin-intoxicated cells. This effect is particularly evident in MCF7 cells. 

The combination of scavenger plus inhibitor pre-treatment did not determine any significant additive protective effect with respect to the single pre-treatment in all the tested cell lines.

## 3. Discussion

In this paper, we demonstrate that in the caudex of *A. heterophylla* is present a galactose-specific lectin, named heterophyllin, which exhibits the characteristics of a type 2 RIP.

SDS-PAGE analysis of the purified lectin showed a single 60 kDa-band, which can be resolved in reducing conditions in two distinct bands of 25 and 33 kDa, corresponding to the A- and B-chain of a type 2 RIP, respectively [36]. Moreover, heterophyllin showed a strong protein synthesis inhibition activity in a cell-free system, with IC_50_ of 2.11 and 0.142 µg/mL in non-reducing and reducing conditions, respectively. These results were comparable to those obtained for other already-characterized Adenia RIPs, which showed IC_50_ values in the same ranges, both in non-reducing and reducing conditions [18,27]. As already observed for lanceolin, stenodactylin, and kirkiin [26,27], heterophyllin did not exhibit a significant deadenylating activity on eukaryotic DNA. This is not surprising, because RIPs have demonstrated high variability in PNAG activity, and all toxic type 2 RIPs possess a PNAG activity significantly lower than type 1 RIPs [12].

Like other Adenia RIPs, heterophyllin agglutinated erythrocytes, showing a minimum agglutinating concentration value very close to that of stenodactylin [26]. All these findings prompt us to confirm that heterophyllin is a new plant toxic lectin belonging to the type 2 RIP family.

One of the major obstacles to the therapeutic use of RIPs is their high immunogenicity, resulting in particular after prolonged therapeutic treatment. To verify if heterophyllin could be used as a drug for the construction of immunotoxins or for local-regional treatment, we tested the cross-reactivity between heterophyllin and some hyperimmune sera directed against different type 1 and type 2 RIPs. Surprisingly, unlike other Adenia RIPs [26,27], heterophyllin poorly cross-reacted with sera against volkensin, stenodactylin, and lanceolin. Interestingly, heterophyllin did not cross-react with sera against ricin and saporin. Since ricin and saporin are the most widely used RIPs in immunotoxin construction [37,38], this prompts us to envisage the use of an immunotoxin containing the heterophyllin A-chain to prolong the therapeutic treatment by replacing the first-used immunotoxins based on ricin A-chain or saporin. In our experiments, heterophyllin showed a low cross-reactivity only with sera against two type 1 RIPs, PAP-R and momordin. This behavior may be due to structural features that need to be investigated in further studies on protein sequence and structure.

Heterophyllin exhibited high cytotoxicity towards different human-derived cell lines, showing EC_50_ values of about 10^−11^ M, after 72 h of intoxication. However, on NB100 cells, the EC_50_ values are close to those obtained with saporin [39] and they are much higher than those obtained with ricin and other Adenia RIPs (2–3 logs) [15,27].

Many experimental findings demonstrated that RIPs induce apoptosis as the main cell death pathway [40,41,42]. Heterophyllin was able to trigger apoptosis, showing elevated Annexin V positivity and negligible necrosis. This represents an advantage in therapy with respect to other cytotoxic agents since the induction of cell death by apoptosis avoids the inflammatory processes related to necrosis activation that, especially in cancer, can lead to important side effects, including the promotion of metastatization.

Increasing experimental evidence is demonstrating that RIPs can induce cell death through different mechanisms in addition to apoptosis. In particular, necroptosis [15,43,44] and mechanisms involving oxidative stress [15,45,46] have been recently described. To investigate the involvement of various cell death mechanisms, experiments were performed by pre-treating cells with scavengers of oxidative stress and with inhibitors of apoptosis and necroptosis. In our experimental conditions, all the scavengers and inhibitors conferred high protection against both heterophyllin and ricin intoxication. Our data suggest a strong involvement of oxidative stress, apoptosis, and necroptosis in the pathogenesis of heterophyllin and ricin intoxication. However, the extent of protection given by scavengers and inhibitors depends on the RIP and cell line. Despite the high rescue of cell viability obtained after the pre-treatment with single agents, the combination of scavenger plus inhibitor conferred neither complete protection nor an additive effect. These results are different from those obtained with stenodactylin on NB100 cells, in which the pre-treatment with scavenger plus inhibitor gave an addictive effect and total protection [15]. Our data suggest that heterophyllin and ricin can induce other mechanisms in addition to oxidative stress, apoptosis, and necroptosis. This represents a fundamental requirement in anti-cancer therapy, since the use of drugs, able to trigger multiple death pathways, can avoid the selection of resistant clones. Further studies will be necessary to identify the involvement of autophagy and/or endoplasmic reticulum stress, which have been already described for some RIPs [16,47,48,49,50,51,52].

## 4. Conclusions

Heterophyllin is a new type 2 RIP, having peculiar immunological and cytotoxic characteristics, which make it different from other already-known type 2 RIPs. These characteristics make heterophyllin a potentially good candidate for therapeutic use, both as a single agent, in loco-regional treatments, and as a toxic component of immunotoxins, for the systemic treatment of tumors or other pathologies. The peculiarities of heterophyllin stimulate further in-depth studies aimed at its possible application in experimental therapies.

## 5. Materials and Methods

### 5.1. Purification and Characterization of Adenia heterophylla Toxic Lectin

*Adenia heterophylla* caudex was purchased from Exotica Botanical Rarities, Erkelenz-Golkrath, Germany, and was kept in the greenhouse of the Botanical Garden of the University of Bologna until use. *A. heterophylla* caudex (460 g) was decorticated and homogenized, as described in [27]. The supernatant (300 mL, containing 650.2 mg of protein) was loaded onto a Sepharose CL-6B column (30 cm height × 2.6 cm diameter) (GE Healthcare, Buckinghamshire, UK), pre-treated with 0.2 M HCl, as described in [27]. After washing with PBS to remove the unbound substances, the lectin proteins, bound to the resin, were eluted stepwise, using 0.5 M galactose in PBS. The protein content of crude extract and not retained material was spectrophotometrically determined, using the Kalb and Bernlohr method [53].

The fractions eluted from Sepharose CL-6B were analyzed by SDS-PAGE on a PhastGel Gradient 4–15%, using the PhastSystem (GE Healthcare, Buckinghamshire, UK). The electrophoretic analysis was performed as described in [26].

### 5.2. Heterophyllin Hemagglutinating and Enzymatic Activities

The hemagglutinating activity was determined in 96-well microtiter plates as described in [26]. The presence or absence of agglutination was visually determined.

The protein synthesis inhibitory activity of heterophyllin was evaluated through a cell-free rabbit reticulocyte lysate system. Experiments were carried out both under non-reducing and reducing conditions, incubating heterophyllin with 1% 2-mercaptoethanol at 37 °C for 30 min. The experiments were conducted as described in [27]. Each experiment was conducted in duplicate and the concentration of RIP causing 50% inhibition of protein synthesis (IC_50_) was calculated by linear regression analysis. Specific activity is expressed as units (U) per mg of protein, where one U is the amount of proteins (in μg) inhibiting 50% protein synthesis in 1 mL of reaction mixture. Total activity was calculated as the specific activity per whole basic-fraction proteins (mg) normalized to the total proteins of the crude extract.

Polynucleotide:adenosine glycosylase activity was spectrophotometrically determined by measuring at 260 nm the adenine release from herring sperm DNA, according to the method reported in [54,55].

### 5.3. Heterophyllin Immunological Properties

ELISA assay was performed in a 96-well plate (Sarstedt, Nümbrecht, Germany), as previously described [26], using 2 μg per well of heterophyllin in 100 μL of 50 mM carbonate buffer pH 9.0, containing 15 mM sodium carbonate and 35 mM sodium bicarbonate. After overnight incubation at 4 °C to allow the RIP adhesion, each well was washed 5 times with 200 µL PBS/Tween (137 mM NaCl, 1.5 mM KH_2_PO_4_, 8 mM Na_2_HPO_4_, 0.05% (*v*/*v*) Tween 20) and 200 µL/well of 0.5 mg/mL bovine serum albumin was added, to saturate the charges of the well. After 1 h incubation at 37 °C and further 5 washes with PBS/Tween, reciprocal serum dilutions (from 1:400 to 1:409,600) were added. The dilutions were prepared in 50 mM lactose, 50 mM mannose, and 50 mM galactose in PBS/Tween. Rabbit antisera against RIPs were obtained as described in [56]. After incubation at 37 °C for 3 h and 5 washes with PBS/Tween, an anti-rabbit secondary antibody conjugated to alkaline phosphatase (Merck, Burlington, MA, USA) was added in 100 μL/well at 1:7000 dilution, and the plate was incubated at 37 °C for 1 h. After further 5 washes with PBS/Tween, 100 μL of 1 mg/mL 4-nitrophenyl phosphate disodium (Merck), dissolved in a buffer containing 1 M diethanolamine, 0.5 M MgCl_2_ × 6H_2_O, and 3 mM NaN_3_, were added. The absorption was measured at 405 nm with the Multiskan EX microtiter plate reader (ThermoLabsystem, Helsinki, Finland).

### 5.4. Heterophyllin Cytotoxicity Mechanisms

The cytotoxicity of heterophyllin was assessed by evaluating both cell viability reduction and apoptosis induction. The human neuroblastoma-derived NB100, the breast carcinoma MCF7, and the bladder carcinoma T24 cell lines were from our departmental cell collection at the Department of Medical and Surgical Sciences. Cells were cultured in complete medium (RPMI-1640, supplemented with 10% (*v*/*v*) heat-inactivated fetal bovine serum, 2 mM L-glutamine, 100 U/mL penicillin G, and 100 µg/mL streptomycin) at 37 °C/5% CO_2_ in a HeraCell Haraeus incubator (Hanau, Germany). The absence of mycoplasma infection was routinely checked in all the tested cell lines.

Cell viability was determined using the CellTiter 96^®^ Aqueous One Solution Cell Proliferation Assay (Promega Corporation, Madison, WI, USA). NB100, T24, and MCF7 cells (3 × 10^3^/well) were seeded in 96-well microtiter plates. After 24 h, cells were incubated with scalar dilutions of heterophyllin (from 1 × 10^−8^ to 1 × 10^−13^ M) or ricin (from 1 × 10^−10^ to 1 × 10^−15^ M). After 72 h of incubation at 37 °C, the medium was removed and 20 µL/well of kit was added. After 1 h of incubation at 37 °C, the absorbance at 492 nm was measured by the microtiter plate reader Multiskan EX (ThermoLab systems, Waltham, MA, USA). Each experiment was carried out in triplicate and the effective concentration of RIP reducing 50% of viability (EC_50_) in cell lines was calculated by linear regression analysis.

Apoptotic cell death was evaluated through flow cytometry analysis using the Annexin V-EGFP/PI detection kit (Biovision, Milpitas, CA, USA). Cells (4 × 10^5^) were seeded in 25 cm^2^ flasks, and after incubation with heterophyllin 10^−9^ M for 24 h, the cells were collected in cytofluorimeter tubes, pelleted at 400× *g* for 5 min, washed twice in cold buffer containing 0.14 M NaCl containing 5 mM sodium phosphate buffer, pH 7.4 (PBS), pelleted and resuspended in 300 μL of binding buffer, containing Annexin V-EGFP (3 μL) and PI (3 μL). After 10 min incubation in the dark at room temperature, cells were analyzed by flow cytometry FACSAria (BD, Franklin Lakes, NJ, USA), using the FACSDiva Software (version 8.0.3, 2013).

Heterophyllin cytotoxicity was also evaluated on NB100, T24, and MCF7 cells pretreated with 100 μM Z-VAD, 100 μM Nec-1, 10 U/mL CAT, and 1 mM NaPyr (the highest concentrations resulting not toxic for NB100, T24 and MCF7 cells in preliminary tests). The reagents were added to cells 3 h before RIP treatment. NB100, T24, and MCF7 cells (5 × 10^3^/well) were seeded in 96-well microtiter plates. The protective effects of Z-VAD, CAT, and Nec-1 were also evaluated in two-by-two combination experiments, using the above-reported concentrations and preincubation times. Subsequently, the cells were treated for 2 h with 10^−9^ M heterophyllin or 10^−11^ M ricin and after one wash with 100 μL/well PBS, cells were further incubated for 24 h in a complete medium. Cell viability was evaluated as described above.

### 5.5. Statistical Analysis

Statistical analyses were conducted using XLSTAT-Pro Software, version 6.1.9, 2003 (Addisoft, Inc., Brooklyn, NY, USA). The results are presented as means ± S.D. of three different experiments. The data were analyzed using ANOVA/Bonferroni test or Mann–Whitney U test. Dunnett’s test was used in addition to ANOVA, when necessary.

## Figures and Tables

**Table 1:** *Adenia heterophylla* lectin purification summary.

Purification Step	Protein (mg/mL)	Total Protein (mg)	Total Protein (%)	IC_50_ (µg/mL) ^1^		Agglutinating Activity (µg/mL) ^2^	Specific Activity (U/mg) ^3^	Total Activity (U)	Yield (%)
				Non-Red.	Red.				
**Raw material**	2.17	650.2	100	42.2	9.15	n.a. ^4^	109.3	71,051	100
**Not retained**	1.39	583.0	89.7	117.0	112.0	n.a. ^4^	8.9	5214	7.34
**Purified protein**	0.173	5.622	0.86	2.11	0.142	43.25	7042	39,595	55.7

^1^ Concentration of protein that inhibits the 50% of protein synthesis in a cell-free system of rabbit reticulocyte lysate. Values were calculated by linear regression analysis. ^2^ Minimum concentrations causing hemagglutination. ^3^ Units of IC_50_ (reducing conditions) per 1 mg of protein. ^4^ Not available.

## Data Availability

The data supporting the findings of this paper are available on request from the corresponding author.
